# Overextension of Nonsetting Calcium Hydroxide in Endodontic Treatment: Literature Review and Case Report

**Published:** 2012-06-01

**Authors:** Arash Shahravan, Shahrzad Jalali, Behrooz Mozaffari, Nasim Pourdamghan

**Affiliations:** 1. Oral and Dental diseases Research center, Kerman University of Medical Sciences, Kerman, Iran; 2. Department of Endodontics, Dental School, Kerman University of Medical Sciences, Kerman, Iran; 3. Department of Oral and Maxillofacial surgery, Kerman University of Medical Sciences, Kerman, Iran

**Keywords:** Calcium Hydroxide, Case Report, Extrusion, Non-Setting, Root Canal Treatment

## Abstract

Premixed non-setting calcium hydroxide (CaOH_2_) paste in pressure syringe system is commonly used in root canal therapy. The aim of this paper is to present a case involving an iatrogenic extrusion of the medicament during endodontic treatment and a literature review of similar reports. The present case demonstrates severe tissue necrosis and other deleterious effects following the extrusion of CaOH_2_ paste beyond root apex. A 21-year old female was referred for endodontic treatment of her maxillary left first premolar. After completion of the canal preparation, root canals were filled by premixed CaOH_2_ paste. In the second appointment, a gingival detachment and an irregular zone of necrosis adjacent to the tooth apex was observed. To treat this complication, a mucoperiosteal flap was raised and the extruded material and necrotic tissues were currettaged and the area sutured. The patient was prescribed antibiotics and followed up at 2 weeks, 6 months and 2 years. Two week follow up showed good soft tissue healing. Two years postoperatively, complete radiographic and clinical healing was observed. We can conclude that the application of CaOH_2_ should be carried out with care and preferably applied free hand or with a lentulo spiral rather than in a pressure syringe.

## Introduction

Calcium hydroxide (CaOH_2_) is a well known root canal medicament [1] and is often used prior to the placement of a permanent root canal filling as a temporary dressing [2]. Non-setting CaOH_2_ paste in pressure syringe system is commonly used in root canal therapy [2]. Although it has been considered as a safe agent [1], a few reports dealt with the negative side effects of CaOH_2_ including bone necrosis and continuing inflammatory response in repaired mechanical perforations, the neurotoxic effect of root canal sealers, cytotoxicity on cell cultures, damaged epithelium with or without a cellular atypia when applied on hamster cheek pouches and cellular damage following early CaOH_2_ dressing of avulsed teeth [3]. Some authors have reported deleterious effects if the material is extruded under a high pressure during endodontic treatment [1][2][4].

Calcium hydroxide paste can result in necrosis and degenerative changes in animal models by intense inflammatory responses [5][6]. It’s pH is around 12 [2]; it has very low solubility at body temperature and will remain in the tissue for considerable time [4] and therefore cannot be considered biocompatible [2].

There are rare reports that have shown unexpected deleterious effects of CaOH_2_ especially in non-setting premixed pressure syringe systems during root canal therapy (Table 1).

**Table 1:**
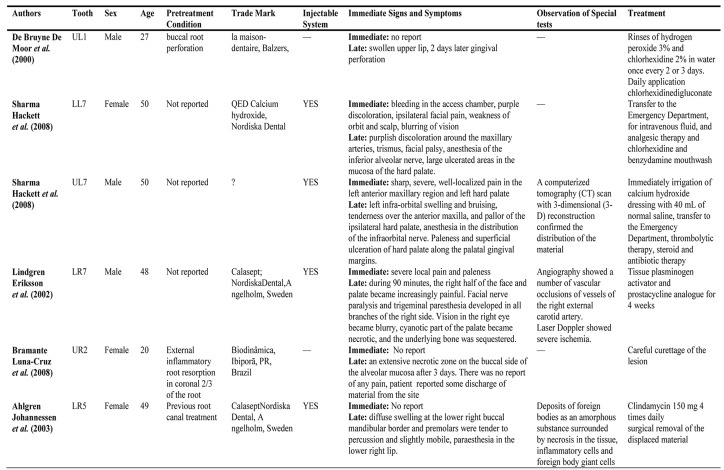
A brief review of reported cases

De Bruyne et al. reported gingival necrosis after extrusion of Ca(OH)_2_ paste (La Maison Dentaire, Balzers, Switzerland) through a root perforation of maxillary central incisor [3] (Table 1).

They treated the necrotic gingival zone with rinses of hydrogen peroxide 3% and chlorhexidine 2% and daily application (BID) of chlorhexidine digluconate 10 mg/g gel and concluded that as long as CaOH_2_ does not come into direct contact with surrounding soft tissues, problems either do not occur or are of a mild transient nature.

Sharma et al. described two severe cases of iatrogenic extrusion of CaOH_2_ (QED CaOH_2_, Nordiska Dental, Angelholm, Sweden) on upper and lower molar tooth causing extensive necrosis in the scalp, skin, and mucosa in the first case and infraorbital nerve paraesthesia and palatal mucosal necrosis in second case (Table 1) [2]. Both patients reported severe pain immediately after CaOH_2_ injection. A computerized tomography (CT) scan with 3-dimensional (3-D) reconstruction in second case confirmed the intravascular distribution of the material. Authors explained that an exposure of CaOH_2_ to blood resulted in crystalline precipitation and the consequent ischemic tissue necrosis. Their patient underwent thrombolytic, steroid and antibiotic therapies to maintain tissue reperfusion, limit inflammatory responses, and prevent infections, respectively. Lindgren et al. reported a case of CaOH_2_ (Calasepts, Nordiska Dental, Angelholm, Sweden) injection into the root of a lower second molar, the inferior alveolar and farther maxillary and external carotid artery, causing necrosis of the ear lobe and superficial necrosis of the cheek skin [1]. When the paste was applied with a syringe in the distal canal, the patient experienced severe local pain. Angiogram showed a number of vascular occlusions in the right external carotid artery branches.

Bramante et al. reported a case of CaOH_2_ therapy for root resorption control in a maxillary lateral incisor (Table 1) [7]. Three days after CaOH_2_ placement (Biodinâmica, Ibiporã, PR, Brazil), an irregular zone of necrosis was observed on buccal mucosa. Careful curettage was performed around the region for removal of necrotic tissue and extruded CaOH_2_; healing was observed at a 15-day follow-up.

Ahlgren et al. showed paraesthesia and changes in surrounding bone after a mishap with CaOH_2_ extrusion (Calasept Nordiska Dental, Angelholm, Sweden) through the apex of a mandibular premolar tooth [4]. They surgically excavated the excessive paste from the spongious bone and after six months, patient was symptom free.

Four cases of seven patients reported moderate to severe pain immediately after CaOH_2_ injection. Blurred vision occurred in two cases, anesthesia or paraesthesia in four, swelling in three, facial palsy or weakness in two, and mucosal ulceration in six cases. In two cases, angiogram or computerized tomography scanning revealed vascular obstruction. In five of the above cases, pressure syringe system was the culprit; however, two cases did not use a pressure syringe system for the application of CaOH_2_.

In this report, we described severe local damages related to CaOH_2_ extrusion from root apices in one patient.

## Case report

### The first session:

**Figure 1:**
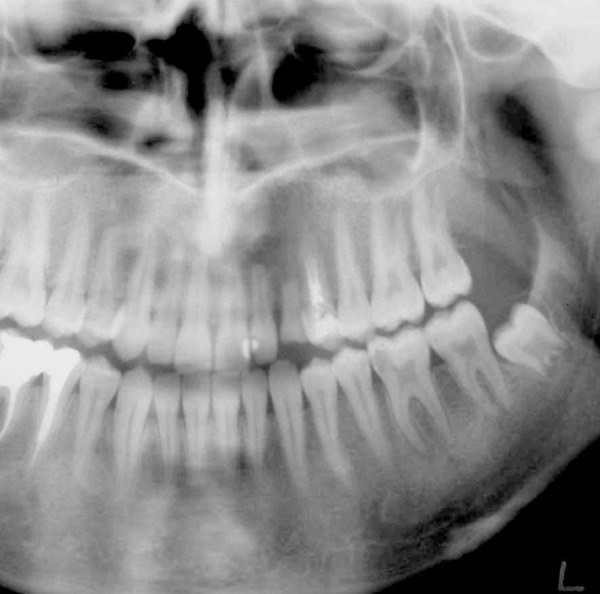
Orthopantomogram showing inadequate root canal filling of maxillary left first premolar

A 21-year old woman was referred by a prosthodontist to receive treatment for her maxillary left first premolar to an endodontist. Examinations indicated the necessity of retreatment due to inadequate root canal preparation/obturation (Figure 1). Her medical history was unremarkable. After local anesthesia infiltration (Lidocaine 2% with epinephrine), access cavity was prepared. Gutta-Percha removal, cleaning and shaping processes were performed by Ni-Ti rotary instruments (Hero 642 Micro Mega) and chlorhexidine digluconate 0.12% irrigant without using any gutta-percha solvent. The working length was determined by an electronic apex locator (Root-Zx) and confirmed by radiography. After completion of the cleaning and shaping of the root canals, CaOH_2_ paste (Calasept; Nordiska Dental AB, Angelholm, Sweden) was inserted into the canal, then access cavity was temporarily filled by Cavit. At the end of the treatment session, the patient complained of moderate pain in that area. The patient was therefore prescribed NSAIDs (400mg Ibuprofen, every 4 hours) and an appointment was made for one week later.

### The second session:

**Figure 2:**
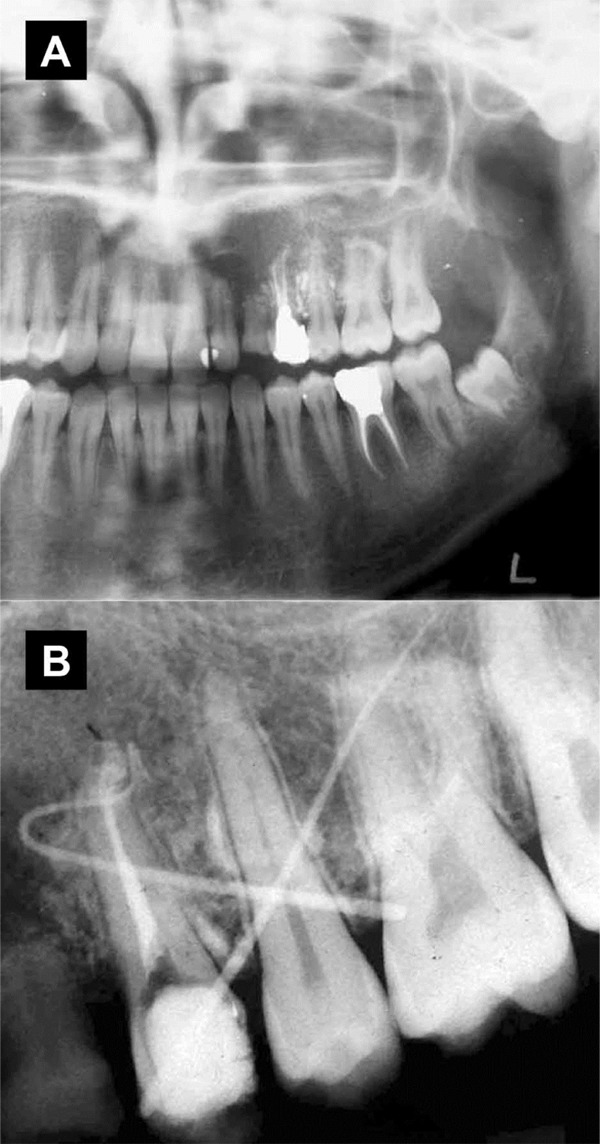
Radiographs showing radiopaque CaOH_2_ paste surrounding root of the premolars (A) and the tracing of gingival detachment by gutta-percha (B)

**Figure 3:**
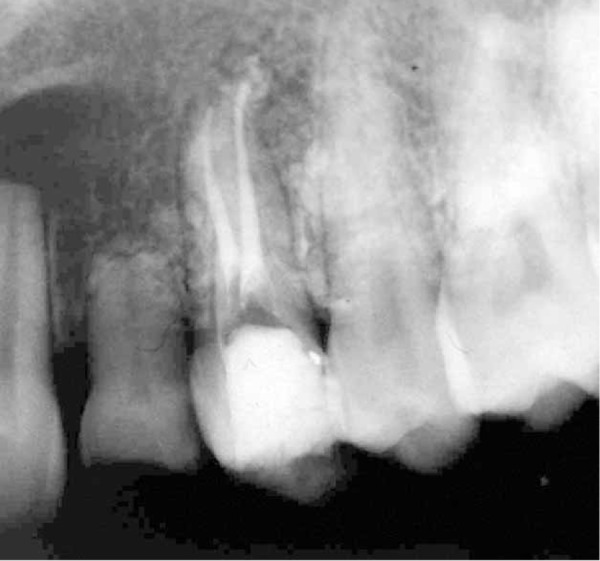
Radiograph showing complete root canal therapy

Clinical examination in the follow-up appointment demonstrated a gingival detachment on the buccal region (from canine to second molar) and an irregular zone of necrosis next to the apex of the first premolar were observed. Radiographs revealed scattered radiopaque material surrounding the region (Figure 2). Permanent root canal filling with gutta-percha was performed with cold lateral compaction technique (Figure 3).

### The third session:

**Figure 4:**
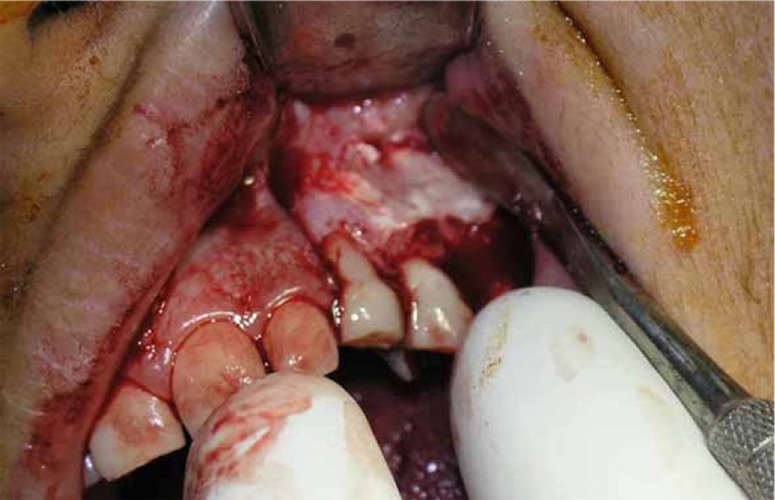
Discolored cortex due to an ischemia and decreased blood supply

**Figure 5:**
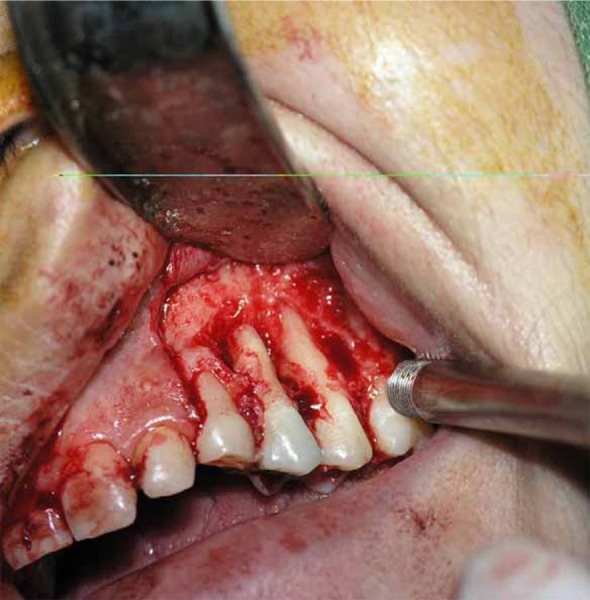
Removal of foreign material and necrotic tissues around the roots

To evaluate the affected area, removal of necrotic tissues and extruded materials, periradicular surgery was performed by an endodontists and an oral and maxillofacial surgeon at a multidisciplinary treatment session. After raising a mucoperiosteal flap from deciduous left canine to second molar with a releasing incision positioned mesial to canine, ischemic and necrotic tissue area and extruded CaOH_2_ was observed (Figure 4). The tissue was curettaged until healthy and bleeding bone was evident (Figure 5). The area was irrigated with saline throughout the procedure. The excavated tissues were immersed in 10% formalin solution for histopathological analysis. After suturing, patient was prescribed Ibuprofen (400mg) and Amoxicillin (500mg, TDS for 5 days) and to return for a review appointment two week later.

### Histopathological evaluation:

**Figure 6:**
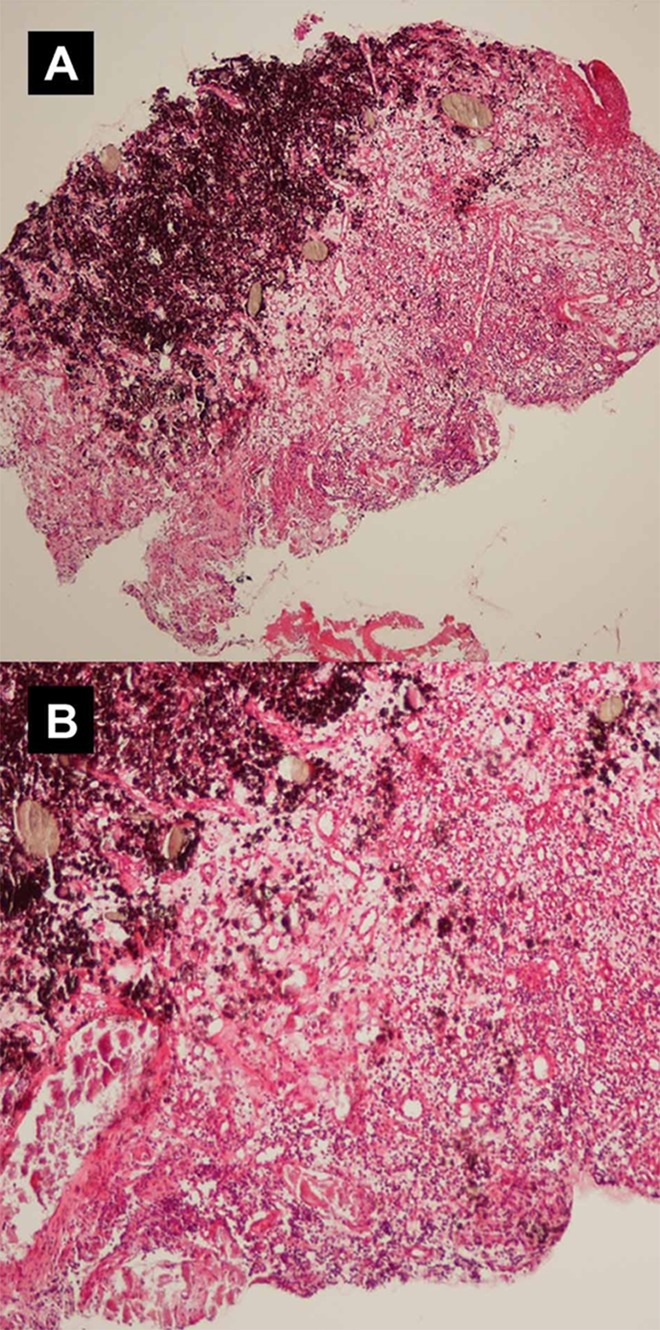
Photomicrographs of biopsy specimen showing foreign material surrounded by necrotic tissues and foreign body reaction (A, B)

**Figure 7:**
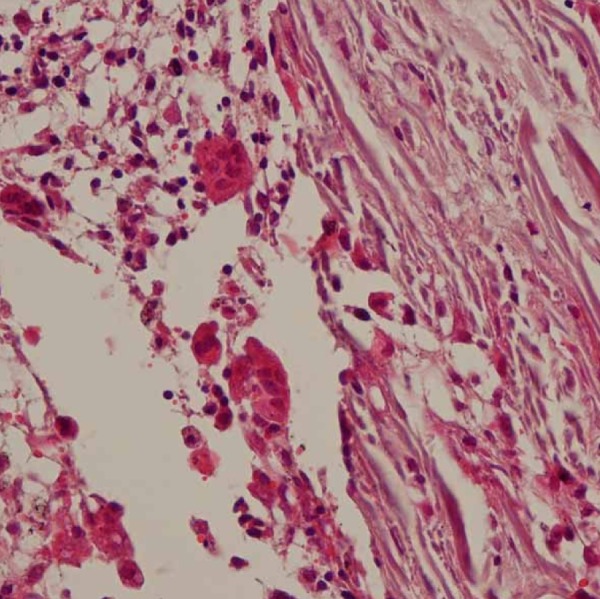
Giant cells and macrophages

The tissue sections were stained with H & E and examined by light microscopy for histopathological changes. Histopathological analysis revealed areas of degenerative changes and necrosis in the tissues in direct contact with the injected paste. Granulomatous tissues containing numerous giant cells and macrophages with engulfed particles in their cytoplasm were also observed in contact with the extruded material. The aggregation of macrophages and giant cells around CaOH_2_ particles in the absence of other inflammatory cells such as neutrophils, lymphocytes, and plasma cells suggests that the paste induced a typical foreign body reaction (Figure 6, Figure 7).

### The first follow up:

**Figure 8:**
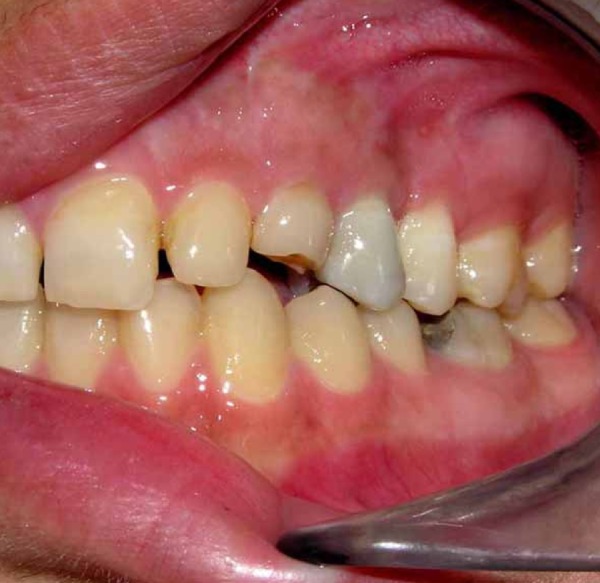
Photograph showing significant soft tissue healing two weeks after suture removal

On the first recall appointment (two weeks after suture removal), complete closure of the lesion with soft tissue was observed (Figure 8).

### Second follow up:

Approximately 6 months later, clinical and radiographic evaluation revealed no signs and symptoms and no lesions in periapical radiograph.

### Last follow up:

**Figure 9:**
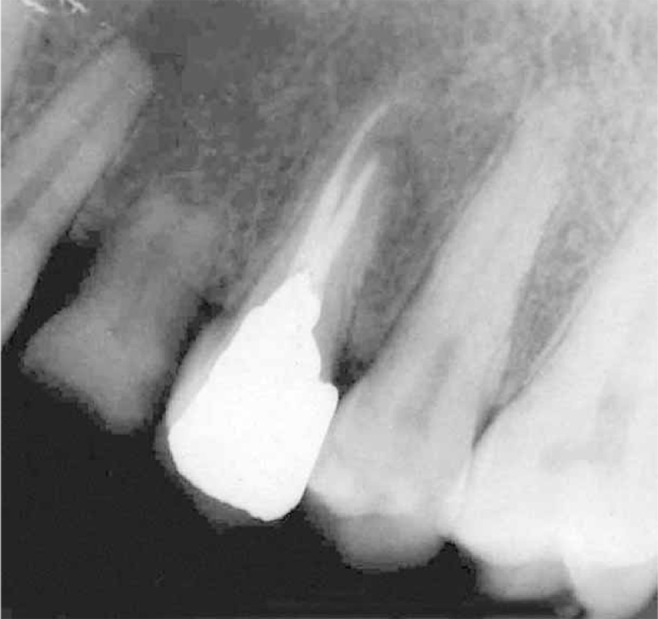
Periapical radiograph showing normal hard tissue after 2 years

Two years later, examination revealed complete clinical and radiographic normalcy with normal pocket depth (Figure 9).

## Discussion

Drug side effects increase the risk for vascular complications due to their physicochemical, pharmacological and galenic-formulation properties. Very acidic or hyperosmolar substances can result in direct tissue necrosis at the site of injection with obliteration of vascular flow [8].

Our histopathological analysis revealed areas of necrosis, and granulomatous tissues containing numerous foreign body giant cells that were similar to some of the previously reported cases [4][7] or animal studies [5][6]. Treatment protocol of our case was similar to some other cases included surgical removal of necrotic tissues and extruded materials [4][7].

It is generally thought that when CaOH_2_ does not come into direct contact with surrounding soft tissues, problems do not either occur or they may occur with a mild transient nature [3]. However, if CaOH_2_ comes into direct contact with the periodontal and gingival tissues, surgical removal is necessary. In two study cases, thrombolytic, steroid and antibiotic therapies were administered to maintain tissue reperfusion, limit inflammatory responses, and prevent infections [2]. Healing period in six reported cases varied from 15 days to six months; in our case, three weeks after surgery, complete closure of the lesion by soft tissue occurred. Six months later, complete clinical and radiographic normalcy were observed. It seems that tissue necrosis maybe due to CaOH_2_ reaching the capillary bed and causing direct tissue toxicity and severe ischemia; in the present case, such findings were limited to the tissues next to the apices.

Typically upon intra-arterial injections, patients can experience immediate pain and tenderness extending from the site of injection to the hand. Ipaktchi et al. [8] reported a case of hand ischemia after intra-arterial injection of water dissolved zolpidem. Angiography revealed no flow in the distal ulnar artery and minimal flow in the palmar arch. Emergent intra-arterial thrombolysis with urokinase was performed and hand perfusion was restored.

Chang et al. described the injection of crushed zolpidem, the identical drug and form of drug administration; in this case, gangrenous loss of several digits was seen despite aggressive therapeutic management and was attributed to microcrystalline cellulose, the drug carrier in zolpidem [9].

The complication that occurred in this case study may be due to binding of the CaOH_2_ syringe to the root canal deep enough to create pressure higher than arterial blood pressure, thereby allowing the paste to travel upstream. It should be mentioned that the CaOH_2_ paste particles are small enough to permeate into capillaries [1] and subsequently obstruct the capillaries mechanically or induce crystalli-zation in the blood, blocking circulation. The extruded medicament may also obstruct the blood circulation by producing thrombi.

The ingredients of readymade CaOH_2_ premixed dressings are variable. For example, Pulpdent Paste contains premixed calcium hydroxide methylcellulose pulpal dressing, whereas 100 grams of Calasept Paste contains 41.07g calcium hydroxide, 8.33g Barium Sulfate and 50.60g Sterile Isotonic Saline Solutions. It has a pH of 12.4 and no methylcellulose carrier.

Instrumentation may develop a traumatic communication facilitating the passage of fluids into the artery. Preparing the canal non-traumatically will reduce the likelihood of extruded endodontic material into the periradicular region. The lentulo spiral is the most effective agent in delivering CaOH_2_ paste to working length and syringe systems are less exact for carrying the filling material [10]. Moreover, there is greater risk of calcium hydroxide extrusion when using pressure syringe systems.

## Conclusions

Caution should be taken when using premixed, pressure syringe CaOH_2_ system in root canal therapy, especially if there is no apical stop in the root apex. We would recommend alternative techniques for applying calcium hydroxide to prevent harmful side effects.

## References

[1] Lindgren P, Eriksson KF, Ringberg A (2002). Severe facial ischemia after endodontic treatment. J Oral Maxillofac Surg.

[2] Sharma S, Hackett R, Webb R, Macpherson D, Wilson A (2008). Severe tissue necrosis following intra-arterial injection of endodontic calcium hydroxide: a case series. Oral Surg Oral Med Oral Pathol Oral Radiol Endod.

[3] De Bruyne MA, De Moor RJ, Raes FM (2000). Necrosis of the gingiva caused by calcium hydroxide: a case report. Int Endod J.

[4] Ahlgren FK, Johannessen AC, Hellem S (2003). Displaced calcium hydroxide paste causing inferior alveolar nerve paraesthesia: report of a case. Oral Surg Oral Med Oral Pathol Oral Radiol Endod.

[5] Nelson Filho P, Silva LA, Leonardo MR, Utrilla LS, Figueiredo F (1999). Connective tissue responses to calcium hydroxide-based root canal medicaments. Int Endod J.

[6] Shimizu T, Kawakami T, Ochiai T, Kurihara S, Hasegawa H (2004). Histopathological evaluation of subcutaneous tissue reaction in mice to a calcium hydroxide paste developed for root canal fillings. J Int Med Res.

[7] Bramante CM, Luna‐Cruz SM, Sipert CR, Bernadineli N, Garcia RB, Moraes IG, Vasconcelos BC (2008). Alveolar mucosa necrosis induced by utilisation of calcium hydroxide as root canal dressing. int dent journal.

[8] Ipaktchi K, Ipaktchi R, Niederbichler AD, Vogt PM, Knobloch K (2008). Unrecognized hand ischemia after intraarterial drug injection: successful management of asuccessful management of a" near miss" event. Patient Saf Surg.

[9] Chang MY, Lin JL (2003). Irreversible ischemic hand following intraarterial injection of zolpidem powder. ClinToxicology.

[10] Sigurdsson A, Stancill R, Madison S (1992). Intracanal placement of Ca (OH)2: a comparison of techniques. J Endod.

